# Supplementary material for: Internal cohesion gradient as a novel mechanism of collective cell migration
Source: PLoS Comput Biol. 2025 Mar 10;21(3):e1012769. doi: 10.1371/journal.pcbi.1012769 (PMC12077783; doi:10.1371/journal.pcbi.1012769)
Supplement: S1 Matlab Code 1 — The code is written using object orientated programming and is required to work in conjunction with S2 Matlab Code 2: Agent.m. (DOCX) [file pcbi.1012769.s001.docx]

Main_Simulation.m

clear

clear all

close all

clc

record = 0; %to create video, set this equal to 1

%decide model type

StandardLinearSolid = 0; %if 1 use SLS, else use Kelvin-Voigt

num_S = 5; %number of cells total

steps = 2000; %how many iterations code runs

iterate = 0; %starts of the for loop for iterations

noiseevery = 15; %how many iterations noise occurs (how often protrusions "form")

plotevery = 15; %how many iterations code creates plot

dt = 0.0125; %timestep

minattract = 200; %value of the smallest spring constant

maxattract = 2000; %value of the largest spring constant

attract = linspace(minattract,maxattract,num_S); %linearly increasing values from min to max

repel = -100; %value for repulsion (this value rarely has an inpact on the code)

etaenvironment = 0; %stokes limit - representing the viscous environemnt

etaspring = 0.5; %represents the viscosity of the cell (should be positive)

mu = 0; %mean value for gaussian noise (average of 0 means equal chance of protrusions in either direction)

sigma = 0.5; %standard deviation for gaussian noise (protrusion distance as a STD)

all = 0; %if this is 1 then all cells move, else only ends move

middlenoise = 4; %this is the value that reduces the noise of the middle cells

%value of 1 means no noise reduction

wall = 0; %this value controls if agents move to the wall (1) or dont move when they hit the wall (0)

overlap = 0; %this value determines the maximum overlap allowed

zone = 5; %max distance for attraction

avg = []; %tracks the average position of whole cluster

position = []; %tracks individual positions of all agents

velocity = []; %tracks individual (initial) velcoities of all agents

acceleration = []; %tracks individual accelerations of all agents

%create spheres

r = 1;

y = 1;

array = [1];

for j = 2:num_S

y(j) = y(j-1) + 2*r;

array = [array j];

end

for i = 1:num_S

S(i) = Agent(0,y(i),r);

S(i).C_attract = attract(i);

S(i).C_repel = repel;

S(i).Zone = zone;

S(i).ALimit = 3;

S(i).V = 0;

S(i).A = 0;

S(i).Jerk = 0;

end

%setup video

if record == 1;

vidfile = VideoWriter('Stochastic Example.mp4','MPEG-4');

open(vidfile);

end

for iterate = 1:steps;

%generate noise

if iterate/noiseevery==round(iterate/noiseevery) %only occurs on noiseevery timesteps

if all == 1 % then move all cells

randarray = array(randperm(length(array)));

for n = randarray

if n == 1

randmax = S(2).Y-S(1).Y-(S(1).R+S(2).R);

randmax = randmax + overlap;

move = mu + sigma .* randn;

if move > randmax

move = wall*randmax;

end

S(1).Y = S(1).Y + move;

elseif n == num_S

randmin = -(S(num_S).Y-S(num_S-1).Y-(S(num_S).R+S(num_S-1).R));

randmin = randmin - overlap;

move = mu + sigma .* randn;

if move < randmin

move = wall*randmin;

end

S(num_S).Y = S(num_S).Y + move;

else

randmin = -(S(n).Y-S(n-1).Y-(S(n).R+S(n-1).R));

randmin = randmin - overlap;

randmax = S(n+1).Y-S(n).Y-(S(n+1).R+S(n).R);

randmax = randmax + overlap;

move = mu + sigma .* randn;

move = move/middlenoise;

if move > randmax

move = wall*randmax;

elseif move < randmin

move = wall*randmin;

end

S(n).Y = S(n).Y + move;

end %if n == 1

end %for n = randarray

else % move ends only

randmax = S(2).Y-S(1).Y-(S(1).R+S(2).R);

randmax = randmax + overlap;

move = mu + sigma .* randn;

if move > randmax

move = wall*randmax;

end

S(1).Y = S(1).Y + move;

randmin = -(S(num_S).Y-S(num_S-1).Y-(S(num_S).R+S(num_S-1).R));

randmin = randmin - overlap;

move = mu + sigma .* randn;

if move < randmin

move = wall*randmin;

end

S(num_S).Y = S(num_S).Y + move;

end %if all == 1

end %if noiseevery

%calculate avearge position

total = 0;

for k = 1:num_S

total = total + S(k).Y;

position(iterate,k) = S(k).Y;

velocity(iterate,k) = S(k).V;

acceleration(iterate,k) = S(k).A;

end

thediff = sum(diff(position));

avg(iterate) = total/num_S;

XVAJ = [position(iterate,:);etaenvironment*velocity(iterate,:);etaenvironment*acceleration(iterate,:)];

yv = [position(iterate,:);etaenvironment*velocity(iterate,:)];

%calculate forces:

for d = 1:num_S-1

[S1F,S2F] = Forces(S(d),S(d+1));

S(d).Force = S(d).Force + S1F;

S(d+1).Force = S(d+1).Force + S2F;

end

fobj = [];

for f = 1:num_S

fobj(f) = S(f).Force(2); % 1D in the y-direction

end

if StandardLinearSolid == 1

k1 = SLS(0,XVAJ,fobj,etaspring);

k2 = SLS(0+dt/2,XVAJ+(dt/2).*k1,fobj,etaspring);

k3 = SLS(0+dt/2,XVAJ+(dt/2).*k2,fobj,etaspring);

k4 = SLS(0+dt,XVAJ+dt.*k3,fobj,etaspring);

XVAJ = XVAJ + (1/6).*dt*(k1 + 2.*k2 + 2.*k3 + k4);

%update positions

for u = 1:num_S

S(u).Y = XVAJ(1,u);

S(u).V = XVAJ(2,u);

S(u).A = XVAJ(3,u);

S(u).Force = 0;

end

else

k1 = Voigt(0,yv,fobj,etaspring);

k2 = Voigt(0+dt/2,yv+(dt/2).*k1,fobj,etaspring);

k3 = Voigt(0+dt/2,yv+(dt/2).*k2,fobj,etaspring);

k4 = Voigt(0+dt,yv+dt.*k3,fobj,etaspring);

yv = yv + (1/6).*dt*(k1 + 2.*k2 + 2.*k3 + k4);

for u = 1:num_S

S(u).Y = yv(1,u);

S(u).V = yv(2,u);

S(u).Force = 0;

end

end %if SLS == 1

%visualization

if iterate/plotevery==round(iterate/plotevery)

subplot(3,5,[1 6 11])

colormap hsv;

cla;

brighten(1);

light;

lighting phong;

hold on;

for a = 1:num_S

Plot(S(a),1);

end

shading interp

axis equal

box on

set(gca,'xtick',[], 'ytick', [])

axis([-2 2 -15 25])

ylabel('position');

hold off;

drawnow

subplot(3,5,[2 3 4 5 7 8 9 10 12 13 14])

plot(iterate,avg(iterate), 'd', "Color", 'k')

hold on

plot(iterate,S(1).Y, 'x', 'Color', 'r')

plot(iterate,S(2).Y, 'x', 'Color', 'b')

plot(iterate,S(3).Y, 'x', 'Color', 'g')

plot(iterate,S(num_S-1).Y, 'x','Color', 'm')

plot(iterate,S(num_S).Y, 'x', 'Color', 'c')

xlabel('time');

if record == 1;

F(iterate) = getframe(gcf);

writeVideo(vidfile,F(iterate));

end

end %if plotevery

end

if record == 1;

close(vidfile)

end

%calculate speed

x = linspace(1,iterate,iterate);

coeff = polyfit(x,avg,1);

y = polyval(coeff,x);

Slope = coeff(1);

figure(2)

plot(avg, 'k', 'linewidth',1)

hold on

plot(x,y, '--k', 'linewidth',2)

plot(position(:,1), 'r')

plot(position(:,2), 'b')

plot(position(:,3), 'g')

plot(position(:,end-1), 'm')

plot(position(:,end), 'c')

xlabel('time'); ylabel('position');

title(['Slope = ',num2str(Slope)],'fontsize',18,'color','k')

zoom on

%SLS Function

function XVAJdot = SLS(t,XVAJ,force,etaspring)

Tmaxwell = 1; %if maxwell time is less than 1 need to reduce dt

eta = -0.75;

XVAJdot(1,:) = XVAJ(2,:);

XVAJdot(2,:) = XVAJ(3,:);

XVAJdot(3,:) = force./Tmaxwell + eta.*XVAJ(2) - (1./Tmaxwell).*XVAJ(3);

end

%Kelvin-Voigt Function

function ydot = Voigt(t,xv,force,etaspring)

ydot(1,:) = xv(2,:);

ydot(2,:) = force - etaspring*xv(2,:);

end

%Force Calculation

function [S1F, S2F] = Forces(S1,S2)

S1Fx = zeros(size(S1.X));

S1Fy = zeros(size(S1.Y));

S2Fx = zeros(size(S2.X));

S2Fy = zeros(size(S2.Y));

for i = 1:length(S1.X)

for j = 1:length(S2.X)

touchdistance = S1.R(i) + S2.R(j);

zonemax = touchdistance + S2.Zone(j);

distance = sqrt(((S2.X(j) - S1.X(i)).^2 + (S2.Y(j)-S1.Y(i)).^2));

attract = [S1.C_attract(i) S2.C_attract(j)];

if distance < zonemax

if distance > touchdistance %stretched/attract

DD = distance - touchdistance;

Dx = ((S2.X(j)-S1.X(i))./(distance)).*DD;

Dy = ((S2.Y(j)-S1.Y(i))./(distance)).*DD;

Fx = (min(attract))*Dx;

Fy = (min(attract))*Dy;

S1Fx(i) = S1Fx(i) + Fx;

S2Fx(j) = S2Fx(j) + (-Fx);

S1Fy(i) = S1Fy(i) + Fy;

S2Fy(j) = S2Fy(j) + (-Fy);

elseif distance < touchdistance %compressed/repel

DD = touchdistance - distance;

Dx = ((S2.X(j)-S1.X(i))./(distance)).*DD;

Dy = ((S2.Y(j)-S1.Y(i))./(distance)).*DD;

Fx = (S2.C_repel(j))*Dx;

Fy = (S2.C_repel(j))*Dy;

S1Fx(i) = S1Fx(i) + Fx;

S2Fx(j) = S2Fx(j) + (-Fx);

S1Fy(i) = S1Fy(i) + Fy;

S2Fy(j) = S2Fy(j) + (-Fy);

end %ifdistance

end %zonemax

end %for j

end %for i

S1F = [S1Fx; S1Fy];

S2F = [S2Fx; S2Fy];

end

[*Published with MATLAB® R2023b*](https://www.mathworks.com/products/matlab/)
