# Supplementary material for: Internal cohesion gradient as a novel mechanism of collective cell migration
Source: PLoS Comput Biol. 2025 Mar 10;21(3):e1012769. doi: 10.1371/journal.pcbi.1012769 (PMC12077783; doi:10.1371/journal.pcbi.1012769)
Supplement: S2 Matlab Code 2 — This code is required to run S1 Matlab Code 1: Main_Simulation.m. (DOCX) [file pcbi.1012769.s002.docx]

Agent.m

classdef Agent < handle

properties

Number %indentifier for agent

X %x position matrix

Y %y position matrix

Z %z position matrix

R %r size matrix

N %number of agents

VolumeMax %Max total volume of cell (determined by first R value assigned)

Volume %individual volumes of each agent (corrected for overlap)

VolumeTotal %total volume of whole object (includes overlaps)

Force %array containing x and y forces for each agent

V %velocity (x')

A %acceleration (same size as force) (x'')

Jerk %the derivative of acceleration (x''')

ALimit %highest allowed value of acceleration

C_attract %cadherin attraction values (will various with agent)

C_repel %cadherin repulsion values

SOverlap %allowed overlap between stem cell agents

Kstretch %spring constant when agents are seperated

Kcompress %spring constant when agents are overlapping

Zone %value determining reach distance for attraction

end

properties (Constant)

zint = 0; %Z position for 3D (always 0 in 2D)

Mass = 1

end

methods

function obj = Agent(x,y,r)

obj.N = 1; %This creates the first agent

obj.X(obj.N) = x;

obj.Y(obj.N) = y;

obj.Z(obj.N) = obj.zint;

obj.R(obj.N) = r;

obj.Force = [0;0];

obj.A = [0;0];

obj.Volume = (4/3)*pi*(r^3);

obj.VolumeMax = (4/3)*pi*(r^3);

end %Agent

function Plot(obj,color)

%Plotting the photoreceptors as spheres

[spherex,spherey,spherez] = sphere(50);

for kk = 1:obj.N

surf(obj.X(kk)+obj.R(kk)*spherex, obj.Y(kk)+obj.R(kk)*spherey, obj.Z(kk)+obj.R(kk)*spherez, color+obj.R(kk)*spherez*0);

end %for kk

end %plot

end %methods

end %classdef

[*Published with MATLAB® R2023b*](https://www.mathworks.com/products/matlab/)
