# Supplementary material for: Internal cohesion gradient as a novel mechanism of collective cell migration
Source: PLoS Comput Biol. 2025 Mar 10;21(3):e1012769. doi: 10.1371/journal.pcbi.1012769 (PMC12077783; doi:10.1371/journal.pcbi.1012769)
Supplement: Attachment — Submitted filename: PLoS_CompBio_Peer_Review_2024.pdf [file pcbi.1012769.s003.pdf]

This manuscript investigates whether cell clusters can generate collective motion via gradients in their individual adhesive ability, even in conditions where they would otherwise move randomly in isolation. The authors motivate their research by noting that cells in a fixed FGF gradient tend to move randomly in isolation but exhibit persistent movement when placed in clusters. Citing research linking FGF sensing to upregulation of cadherins (a membrane protein associated with higher cell-cell adhesion), the authors develop a 1D mathematical model in which cells are represented as point particles connected by spring-dashpot systems. The authors apply a gradient by having upper cells in a "cluster" respond to perturbations in their equilibrium separation distance more strongly than lower cells. The authors show that perturbing the first and last cell in a cluster periodically does not lead to persistent motion of the cluster center of mass, but that perturbing the upper and lower cell positions at stochastic time intervals and random amplitudes does lead to a biased trajectory. The authors conclude by presenting *in silico* results in which the cluster center of mass does not seem to change much when cells in the cluster center are perturbed. The authors claim that this model demonstrates how noise and asymmetries within cell clusters can impact collective movement.

This paper is not ready for publication. The evidence to support the authors' claims of persistent movement is weak, as the length and time scales at which they distinguish between persistent and random migration are too small. Additionally, it appears from the results that the effect of the adhesive gradient in the model is very small when compared with the *in vitro* trajectories presented in the Introduction section. The authors do not comment on alternative drivers of persistent collective cell migration, for example cell-induced chemotactic gradients which have *in vitro* and *in silico* support (Tweedy et al., Science, 2020). There are also several paragraphs which were confusing in this reviewer's opinion and should be revised in a future draft. The authors should consider upscaling their mathematical model to higher dimensions (either 2D or 3D) to represent cells more faithfully in *in vitro* and *in vivo* settings.

Below please find a list of major and minor comments that should be addressed.

<u>List of Major Comments:</u>

1. Figure 1: The length scales in panel (b), which are purported to model persistent trajectories, appear to be the same as those in panel (a), which are claimed to describe random migration. Additionally, the blue and green curves of the two panels appear to travel the same distance in (what I assume to be) the same period. If movement in panel (b) is persistent, as the authors claim, there should be more evident differences between the two plots. It would be more informative to plot over longer time periods, and to show the mean squared displacement, as both are more traditionally done to distinguish between ballistic (persistent) and random movement. Furthermore, it is unclear how the curves have been generated, which creates a misleading impression of the results.

2. Line 62: The authors do not discuss or rule out alternative explanations for why cells migrate more persistently as a collective than as individuals. Relatively recent work (Tweedy et al., Science, 2020) has demonstrated that cell-induced chemotaxis, in which cells create their own chemotactic gradient by consuming or degrading a chemoattractant, can cause groups of cells to move in otherwise uniform environments. Furthermore, the

mechanism of chemotaxis is well established to be a particularly effective means by which cells can travel. The authors should argue, or show evidence, that chemotaxis and related mechanisms do not explain the *in vitro* results from Figure 1 and explain why the mechanism proposed in this article would be non-negligible in comparison to these effects.

3. Line 87: Can the authors explain why they have chosen to work with a 1D model, given that the *in vitro* clusters are likely in 2D or 3D? I agree that a 1D model is necessary for building intuition, but 2D results would be more helpful in demonstrating that the noise-induced persistent motion is not an artefact of a lower-dimensional system. Additionally, a 2D model would be more representative of the underlying biology the authors are trying to describe.

4. Figure 6: What do the solid lines in the figures correspond to? Also, the length scales of the blue and red lines don't seem too different from each other, which leads me to conclude that persistent and random migration cannot be distinguished at this scale. Plotting the mean squared displacement of the center of mass over longer time periods may be more informative.

5. Figure 6: There appears to be a slight drift in the center of mass for the blue and red curves, but is this effect significant compared to the length scales shown in Figure 1? The *in silico* model created in this manuscript can show how adhesive gradients contribute to forward collective motion (at least in 1D), but 2D experiments would be more persuasive and could shed light on whether this mechanism could be as important to collective movement as cell-induced chemotaxis.

<u>List of Minor Comments:</u>

1. Line 23: There appears to be a typo here (*E. coli.* rather than *e.coli*).

2. Lines 63-65: The reference to Noether's theorem requires more explanation. The "symmetries" in that theorem refer to symmetries in the Lagrangian, which is related to the energy functional, and not necessarily to geometric symmetries. To this reviewer, it seems that the geometric symmetry of a cluster has been conflated with a symmetry in the Lagrangian, which I do not think is true in general. Can the authors explain in more detail the connection they are trying to make?

3. Line 74: I believe a comma is needed after "e.g.".

4. Line 89: Calling the cells spherical is misleading since the model is in 1D. A better description may be "point particles".

5. Lines 121-122: A potential issue with using third order models is that they can admit more than one solution in certain cases, if only the initial positions and velocities are specified. Can the authors describe here how they ensure their problem is well-posed?

6. Lines 136-139: It is unclear from this sentence how the mechanisms are being altered and how they relate to the model parameters shown in Figure 2. More details should be provided here.

7. Figure 3: Can the authors explain why the cells appear to be roughly equidistant in panels (b) and (c), while they are more extended in panel (a)?

8. Line 195: The second half of this sentence merits further discussion. In what way does this perturbation reflect a positive feedback loop?

9. Line 278: The mean cluster speed corresponds to which parameter in the mathematical model?

10. Line 286: There appears to be a comma missing here.

11. Line 291: More details should be provided here. What did the authors change in their simulations to achieve computational damping?

12. Figure 7: For the inset panels, does the orange line correspond to the predictions of Eqn. [6] as well?

13. Lines 325-329: Could another source of error be that, in high noise environments, the authors have exited the conditions under which the assumptions underlying Eqn. [6] are fulfilled?

14. Figure 8: Could an alternative explanation be that, because perturbations beyond a cell-cell distance are not allowed, cells in the center are not actually perturbed at all because they are too close to their neighbors, and you reduce effectively to the case presented in Figure 3?

15. Lines 450-451: Did the authors correct for their use of multiple comparisons, for example by performing a Bonferroni correction? If not, can they explain why?
