# Supplementary material for: Internal cohesion gradient as a novel mechanism of collective cell migration
Source: PLoS Comput Biol. 2025 Mar 10;21(3):e1012769. doi: 10.1371/journal.pcbi.1012769 (PMC12077783; doi:10.1371/journal.pcbi.1012769)
Supplement: Attachment — Submitted filename: PLOS - Reply to Reviewers.docx [file pcbi.1012769.s006.docx]

**Maribel Vazquez, Sc.D.**

**Professor of Biomedical Engineering**

**599 Taylor Road, BME-219**

**Piscataway, NJ 08854-5610**

<https://www.vazlab.org/>

September 28, 2024

Dr. Feilim Mac Gabhann and Dr. Jason Papin

Editors-in-chief, PLOS Computational Biology

1265 Battery Street, Suite 200

San Francisco, CA 94111

United States

Dear Dr. Gabhann and Dr. Papin,

I write to attach author revisions to the manuscript, ‘***Internal Cohesion Gradient as a Novel Mechanism of Collective Cell Migration.***’ PCOMPBIOL-D-24-00775. The reviewers’ remarks have helped us greatly enhance this manuscript. Each reviewer comment is addressed point-by-point below and highlighted in red text within the revised manuscript.

Please advise if we can provide any further information to aid in the review process.

Thank you.

Sincerely,

____________________________

Dr. Maribel Vazquez

Professor and Principal Investigator

Department of Biomedical Engineering

Rutgers University, The State University of New Jersey

599 Taylor Road, BME-219

Piscataway, NJ 08854-5610

Office: 848.445.6568 Fax: 732.445.3753

<https://www.vazlab.org/>

---------------------------------------------------------------------------------------------------------------------

**REVIEWER #1**

***Reviewer remark:***

*1.1. Figure 1: The length scales in panel (b), which are purported to model persistent trajectories, appear to be the same as those in panel (a), which are claimed to describe random migration. Additionally, the blue and green curves of the two panels appear to travel the same distance in (what I assume to be) the same period. If movement in panel (b) is persistent, as the authors claim, there should be more evident differences between the two plots. It would be more informative to plot over longer time periods, and to show the mean squared displacement, as both are more traditionally done to distinguish between ballistic (persistent) and random movement. Furthermore, it is unclear how the curves have been generated, which creates a misleading impression of the results.*

***Author Reply:***

We thank the reviewer for this remark and have updated the caption of Fig. 1 to address these issues. To clarify, the data in Fig. 1 are reproduced from previous experiments (Pena 2019/Markey 2022) and show that clusters of cells migrate in a directed manner, as highlighted by the consistently upward trajectories for cell clusters, while individual cells migrate erratically. We agree that repeating the experiments for longer times would be useful, but this is beyond the scope of the present, computational biology, manuscript and existing experimental data aren’t adequate to produce meaningful MSD analysis. For this reason, we include MSD for our simulation (described in 3^rd^ reply following). Moreover, in the associated papers that we cite (Pena 2019/Markey 2022), Chemotactic Index (CI > 0.5) was used to determine directional migration, where CI for individual cells was 0.22 ± 0.2 and for small clusters 0.61 ± 0.15. The curves are generated from a cubic spline fit to the data points, which we now clarify in the caption.

The following text has been revised in the caption of Figure 1:

***Figure 1 – Experimental migration trajectories*** *toward higher FGF concentration (indicated by the black arrow) for single cells (left) and clusters of ~10 Drosophila retinal progenitor cells in microchannels prepared with nearly steady FGF gradient (upward in plot orientation). A chemotactic index (CI) greater than 0.5 indicates positive directional migration along concentration gradients (17,19). Small clusters of cells tend to migrate toward the higher FGF concertation (Average CI = 0.61 ± 0.15) while individual cells appear to migrate more randomly (Average CI = 0.22 ± 0.20). Each plot shows 3 trials (from Pena 2019 (17)); distances traveled are normalized to the number of cells in the cluster. The curves are cubic splines to aid the eye.*

***Reviewer remark:***

*1.2. Line 62: The authors do not discuss or rule out alternative explanations for why cells migrate more persistently as a collective than as individuals. Relatively recent work (Tweedy et al., Science, 2020) has demonstrated that cell-induced chemotaxis, in which cells create their own chemotactic gradient by consuming or degrading a chemoattractant, can cause groups of cells to move in otherwise uniform environments. Furthermore, the mechanism of chemotaxis is well established to be a particularly effective means by which cells can travel. The authors should argue, or show evidence, that chemotaxis and related mechanisms do not explain the in vitro results from Figure 1 and explain why the mechanism proposed in this article would be non-negligible in comparison to these effects.*

***Author Reply:***

We again thank the reviewer for this feedback. The simulations done in this paper are based on previous studies conducted on retinal progenitor cells which show that individual cells do not chemotax, but rather migrate as groups of cells. Moreover, the data was measured in ultra-low concentration gradients that suppress cell ability to create their own local gradient by consuming/degrading the surrounding chemoattractant field. The present manuscript is intended to propose and computationally test a new mechanism in which net chemotaxis of clusters is generated by internal cell-cell interactions. We have revised the text to clarify on this point.

The following text has been revised starting on line 70:

Clinically speaking, the goal of intentionally directing progenitors to desired targets (e.g. to regions of a damaged retina), relies on establishing what factors affect migration and how the factors function. While many studies have demonstrated cell-induced chemotaxis in individual cells (22-24), our studies indicate that individual RPCs do not chemotax without influence from neighboring cells (17, 19, 25). Moreover, the data was measured in ultra-low concentration gradients that suppress cell ability to create their own local gradient by consuming/degrading the surrounding chemoattractant field. The underlying mechanisms by which a net external force is produced by interactions between individual cells – that do not individually exhibit directional migration – challenge our understanding (26).

***Reviewer remark:***

*1.3. Line 87: Can the authors explain why they have chosen to work with a 1D model, given that the in vitro clusters are likely in 2D or 3D? I agree that a 1D model is necessary for building intuition, but 2D results would be more helpful in demonstrating that the noise-induced persistent motion is not an artefact of a lower-dimensional system. Additionally, a 2D model would be more representative of the underlying biology the authors are trying to describe.*

***Author Reply:***

We agree that 2D and 3D models would be valuable, however our goal of this present manuscript is to provide a mechanism for the unexpected collective motion of clusters in the simplest context. In addition, the growth factor gradients that drive collective migration primarily develop in 1D, which enables direct comparison of the computational model with in vitro models. Future work will involve the development of 2 and 3D models.

The following text has been revised on line 109:

We use a one-dimensional agent-based model to explore the mechanical effects of differential cohesion strength across a cluster of cells on the cluster’s ability to collectively migrate. The decision to use a 1D model is motivated by growth factor gradients developing in one direction and enables direct comparison between *in-silico* and *in-vitro* models.

***Reviewer remark:***

*1.4. Figure 6: What do the solid lines in the figures correspond to? Also, the length scales of the blue and red lines don't seem too different from each other, which leads me to conclude that persistent and random migration cannot be distinguished at this scale. Plotting the mean squared displacement of the center of mass over longer time periods may be more informative.*

***Author Reply:***

The solid lines in Figure 6a correspond to the individual cell positions at that point in time. The straight lines in Figure 6b are least squares fits to the data; the other, more jagged, lines are average cluster positions at successive times. The spatial scale shown in the graph is relative to the radius of the cells. To address the concern about the length scales not appearing different from each other, we have conducted a mean squared displacement analysis on our simulations (now included as Fig. 6(c)) to confirm that clusters without an internal gradient move diffusively while clusters with a gradient diffuse more directionally.

Panel (c) has been added to Fig. 6, and the figure caption has been revised as follows:

***Figure 6 – Simulations of 5-cell cluster with Gaussian fluctuations.*** *(a) Outermost cells, N_1_ and N_5,_ experience displacements according to Gaussian fluctuations. The colored lines show the subsequent positions of each cell within the cluster. Note the upper 4 cells migrate consistently upward, toward the higher internal cohesion gradient, while the lower cell wanders almost independently. (b) Comparison between centers of mass subject to regular extensions (gray, from Fig. 3), Gaussian fluctuations without a cohesion gradient (blue), and Gaussian fluctuations with a gradient (red). Solid lines indicate the average cluster position at successive times, and the dotted line are the least squares fit to the data. Only the case with the gradient migrates persistently, as shown by the positive slope (red dotted line). Key parameters are as follows: gradient strength = 10, cluster size = 5, protrusion amplitude = 1, protrusion period = 15. (c) Mean Squared Displacement analysis of N = 100 simulations of cell clusters without a gradient (blue) and with a gradient (red).*

Additionally, the following text has been revised on line 340 :

Fig 6(c) includes a Mean Squared Displacement (MSD) analysis on 100 simulations. Migrating clusters without an internal cohesion gradient exhibit a linear trend indicating a diffusive or random migration pattern (R^2^ = 0.997), while clusters with an internal gradient exhibit a quadratic trend indicating directed motion (R^2^ = 0.999).

***Reviewer remark:***

*1.5. Figure 6: There appears to be a slight drift in the center of mass for the blue and red curves, but is this effect significant compared to the length scales shown in Figure 1? The in silico model created in this manuscript can show how adhesive gradients contribute to forward collective motion (at least in 1D), but 2D experiments would be more persuasive and could shed light on whether this mechanism could be as important to collective movement as cell-induced chemotaxis.*

***Author Reply:***

The blue and red solid lines in Figure 6 correspond to the average position of the whole cluster at that particular timestep and the dashed line is the mean cluster speed. All the lines describe different scenarios of cluster migration, whereas in Figure 1 we compare individual cell migration to cluster migration. In addition, the data from Figure 1 were extracted from 2D data, but the directional effects are more significant along the y-dimension, as the concentration gradient developed along the y direction (See Pena 2019 for further details).

As mentioned previously, the following text has been revised in the caption of Figure 1:

***Figure 1 – Experimental migration trajectories*** *toward higher FGF concentration (indicated by the black arrow) for single cells (left) and clusters of ~10 Drosophila retinal progenitor cells in microchannels prepared with nearly steady FGF gradient (upward in plot orientation). A chemotactic index (CI) greater than 0.5 indicates positive directional migration along concentration gradients (17,19). Small clusters of cells tend to migrate toward the higher FGF concertation (Average CI = 0.61 ± 0.15) while individual cells appear to migrate more randomly (Average CI = 0.22 ± 0.20). Each plot shows 3 trials (from Pena 2019 (17)); distances traveled are normalized to the number of cells in the cluster. The curves are cubic splines to aid the eye.*

***Reviewer remark:***

*1.6. Line 23: There appears to be a typo here (E. coli. rather than e.coli).*

***Author Reply:***

We thank the reviewer for their careful reading; corrected in the revised manuscript.

The following text has been revised on line 34:

At the oldest end of this range, millions of E. coli cooperate to produce intricate swarming behaviors (2),

***Reviewer remark:***

*1.7. Lines 63-65: The reference to Noether's theorem requires more explanation. The "symmetries" in that theorem refer to symmetries in the Lagrangian, which is related to the energy functional, and not necessarily to geometric symmetries. To this reviewer, it seems that the geometric symmetry of a cluster has been conflated with a symmetry in the Lagrangian, which I do not think is true in general. Can the authors explain in more detail the connection they are trying to make?*

***Author Reply:***

The authors thank the reviewer for this remark. In this case we are referring to the asymmetry of the potential energy from the strength of the cohesive bonds between individual cells, not necessarily a geometrical asymmetry. This is important to Biophysicists: we recognize that Noether’s theorem may seem specialized in biology and engineering, but it is foundational in Physics, underlies every conservation law, and so is essential to the mechanism described in this manuscript. We have revised the text to add clarity on the intended connection.

The following text has been revised on line 82:

This is informative since Noether’s theorem (26, 27) dictates that symmetries (here of cohesive potential energies) are always associated with conservation laws (here of forward momentum), and so our task is to analyze how asymmetries of internal forces can generate a net external force.

***Reviewer remark:***

*1.8. Line 74: I believe a comma is needed after "e.g.".*

***Author Reply:***

We again thank the reviewer for their careful reading.

The following text has been revised on line 93:

We utilize Agent-Based Modeling (ABM), a first-principles approach that defines cells as autonomous “agents” that follow a set of “rules” (e.g., Newton’s laws of motion) based on known mechanisms or observed behaviors.

***Reviewer remark:***

*1.9. Line 89: Calling the cells spherical is misleading since the model is in 1D. A better description may be "point particles".*

***Author Reply:***

We have changed the text to clarify the cellular representation. To be clear, the computational model is 3D, and the cells are actually spheres: this permits future extension of our analysis to 2D and 3D. In the present manuscript, the spheres are constrained to a line to represent 1D migration.

The following text has been revised on line 112:

We represent individual cells as spheres arranged in a line constrained to a planar surface, where each sphere is mechanically linked to its nearest neighbors by damped springs in one of two configurations:

***Reviewer remark:***

*1.10. Lines 121-122: A potential issue with using third order models is that they can admit more than one solution in certain cases, if only the initial positions and velocities are specified. Can the authors describe here how they ensure their problem is well-posed?*

***Author Reply:***

For the Kelvin-Voigt model, which is a 2^nd^ order equation, only the initial positions and velocities are specified. However, for the Standard Linear Solid Model, which is 3^rd^ order, the initial positions, velocities, and accelerations are specified. This is stated in the methods section (lines 426/427).

The following text has been revised on line 146:

Although Eq. [3] is third order, we find that the combined internal and external damping effectively limits numerical instabilities, additional details provided in the Methods section.

The following text has been revised on line 492:

Each agent is assigned an initial position ($x,y)$ such that they are linearly configured with edges just touching their nearest neighbors and an initial velocity $(\dot{x})$ and/or acceleration $\left( \ddot{x} \right)=0$.

***Reviewer remark:***

*1.11. Lines 136-139: It is unclear from this sentence how the mechanisms are being altered and how they relate to the model parameters shown in Figure 2. More details should be provided here.*

***Author Reply:***

The mechanism we are highlighting in this sentence is the inclusion of stochasticity. Simply put, we are stating that a cohesion gradient alone is not sufficient for directional migration, cell must be migrating stochastically as well. This is further explained by figure 3. We have revised the text to add clarity.

The following text has been revised starting on line 165:

We emphasize that although each cellular agent exhibits a different tensile modulus toward the front and the back of the cluster, it is not evident that this difference in *internal* modulus alone will produce a net *external* force, affecting transport of the cluster. Indeed, as we will show through detailed simulations (Fig. 3 and Fig. 7), net chemotaxis only occurs under a limited set of conditions. Interestingly, we find first that one of these conditions is that the cells must migrate stochastically. That is, it is known that most cell types migrate in either using adhesion -dependent (mesenchymal) (50) or -independent (amoeboid) mechanisms (51, 52). However, if migration is deterministically activated (i.e., cells move in alternating inward and outward directions periodically in time), no collective motion of clusters is seen in our simulations. On the other hand, if migration of individual cells is invoked stochastically (i.e. cells moving in either direction randomly), persistent forward motion of clusters emerges.

***Reviewer remark:***

*1.12. Figure 3: Can the authors explain why the cells appear to be roughly equidistant in panels (b) and (c), while they are more extended in panel (a)?*

***Author Reply:***

In panel a, the outermost cells only extend outwards, while in panels b and c the outermost cells oscillate both inward and outward. We have clarified this point in the text.

The following text has been revised in the caption of Figure 3:

***Figure 3 – Simulations of 5-cell clusters with intercellular cohesion gradient.*** *(a) The outermost cells, N_1_ & N_5_, experience an outward extension of the same distance at the same time, and each colored line shows subsequent positions. The periodic extension causes the cluster to elongate until it reaches a natural equilibrium state. The black dashed line shows the center of mass, which remains stationary. (b) The same simulation in which the outermost cells move both up and down in phase, and (c) the same in which the outermost cells move in and out in phase. As expected, in all cases the center of mass remains stationary except for small transient oscillations. Key parameters are as follows: gradient strength (ratio of strongest to weakest cohesion strengths) = 10, cluster size = 5, protrusion amplitude (length of protrusions) = 1, protrusion period (frequency of protrusions) = 15. Protrusion amplitude is a fixed value in this simulation, whereas all other simulations have a variable amplitude using a Gaussian whose standard deviation is the specified protrusion amplitude.*

***Reviewer remark:***

*1.13. Line 195: The second half of this sentence merits further discussion. In what way does this perturbation reflect a positive feedback loop?*

***Author Reply:***

We thank the reviewer for this question. The study the authors are referring to describes a positive molecular feedback loop that occurs at the unconstrainted edge of cells, giving space for initial protrusion formation, which in turn activates a molecular signaling pathway that promotes further protrusion formation (see ref 53).

The following text has been revised on line 234:

This allows direct comparison with Fig. 3 and agrees with recent findings that leader cell specification emerges from the existence of an unconstrained free edge which enables extended protrusions formation. This initiates a positive molecular feedback loop that promotes further protrusion formations (53).

***Reviewer remark:***

*1.14. Line 278: The mean cluster speed corresponds to which parameter in the mathematical model?*

***Author Reply:***

The mean cluster speed is controlled primarily by the internal cohesion strength gradient along with the intrinsic cellular stochasticity (which we refer to as the protrusion period and protrusion amplitude throughout the paper). We further explore how the parameters impact the mean cluster speed in Figure 7 of the paper. We have revised the text to add clarity.

The following text has been revised on line 336:

If the outermost cells wander stochastically without an internal cohesion gradient, the cluster will simply diffuse, equally likely in either direction, with a mean cluster speed of $(-0.1\pm0.1)\cdot{10}^{-3}$ radii per timestep $(mr/\tau$ for brevity), as shown in blue in Fig. 6(b). When we include a cohesion gradient along with stochastic motion, however, we consistently see significant speeds toward the more cohesive end of the cluster as shown in red in Fig. 6(b), where the mean cluster speed is $\left( 2.0\pm0.1 \right)$ $mr/\tau$. Fig 6(c) includes a Mean Squared Displacement (MSD) analysis on 100 simulations. Migrating clusters without an internal cohesion gradient exhibit a linear trend indicating a diffusive or random migration pattern (R^2^ = 0.997), while clusters with an internal gradient exhibit a quadratic trend indicating directed motion (R^2^ = 0.999) (57). These results taken together indicate that a cohesion gradient, when combined with intrinsic cellular stochasticity (described by the protrusion amplitude and period), generate a mechanism for directional migration. We further explore the impact of these key parameters on mean cluster speed in the next section.

***Reviewer remark:***

*1.15. Line 286: There appears to be a comma missing here.*

***Author Reply:***

Thanks once more for this correction. A comma has been added after number of cells.

The following text has been revised on line 351:

We test these predictions by varying the equation’s three available parameters: distance, Δ, between leading neighbors (related to the cohesion gradient strength), number of cells, 𝑁, in a cluster the mean extension distance (proportional to the Gaussian standard deviation, 𝜎), and the period, 𝑇, of extending protrusions.

***Reviewer remark:***

*1.16. Line 291: More details should be provided here. What did the authors change in their simulations to achieve computational damping?*

***Author Reply:***

To achieve computational damping, the velocities were reduced by a constant percentage after every timestep, depending on the model used. This produces exponential decay, as occurs in viscous damping.

The following text has been revised on line 358:

All simulations are computationally damped to mimic a low Reynolds number cellular environment. To achieve this computational damping, we consider the Stokes limit for KV simulations (i.e. we reduce all velocities by 100% after every timestep). SLS simulations include internal viscous creep which creates an additional damping, so we reduce velocities slightly less (by 25%) every timestep for those simulations. This produces exponential decay, as occurs in viscous damping.

***Reviewer remark:***

*1.17. Figure 7: For the inset panels, does the orange line correspond to the predictions of Eqn. [6] as well?*

***Author Reply:***

Yes, Kelvin-Voigt data also correspond to the predictions of Eq. 6. We note that the parameters used to fit Eq. 6 is different for Kelvin-Voigt and Standard Linear Solid. We opted not to plot Eq. 6 in the figure to reduce clutter in an already busy figure, however, we have revised the text to add this clarification.

The following text has been revised on line 388:

Evidently, data from both models are well fit by the $1/N$ prediction from Eq. [6] using their respective parameters.

***Reviewer remark:***

*1.18. Lines 325-329: Could another source of error be that, in high noise environments, the authors have exited the conditions under which the assumptions underlying Eqn. [6] are fulfilled?*

***Author Reply:***

We thank the reviewer for this thought-provoking comment. While we cannot be sure that the reason why the fit worsens in higher noise environments, we argue that it makes sense that in higher noise environments, more collision events would naturally occur between the outermost cells and their neighbors, and these collisions can either help the cluster move or hinder it depending on the direction of the collision. One of the assumptions made to derive Equation 6 is that cells cannot overlap with another cell, and even with higher noise we maintain that rule in the simulations. We have added some text to clarify these assumptions further.

The following text has been added starting on line 393:

Fig. 7(c) shows that the cluster speed grows monotonically as predicted by Eq. [6], but data show greater variation in speed with greater extension length, as exhibited both by longer violin plots at larger extension amplitudes and by standard error bars. We argue that in higher noise environments, more collision events would naturally occur between the outermost cells and their neighbors, and these collisions can either help the cluster move or hinder it depending on the direction of the collision. One of the assumptions made to derive Eq. [6] is that cells cannot overlap with another cell, and even with higher noise we maintain that rule in the simulations. Since the extension amplitude is governed by noise – i.e. by Gaussian standard deviation, $\sigma$, and consequently the fit to Eq. [6] worsens at larger extension amplitudes.

***Reviewer remark:***

*1.19. Figure 8: Could an alternative explanation be that, because perturbations beyond a cell-cell distance are not allowed, cells in the center are not actually perturbed at all because they are too close to their neighbors, and you reduce effectively to the case presented in Figure 3?*

***Author Reply:***

The reviewer raises a thoughtful point that troubled us for some time. Ultimately, in all simulations we found that deterministic forcing (Fig. 3) never produces collective migration, under any conditions, while stochastic motion (e.g. Fig. 8) can. We agree that it is also true that when all cells extend, a state without effective motion of inner cells arises; nevertheless, we cannot produce collective motion without stochastic forcing.

This finding is not entirely intuitive: we initially expected that a simple free-body analysis would produce a net force, but our observation is that this does not occur, and only by stochastic motion do cell collections move. It’s possible that something akin to stochastic resonance is occurring, but further investigation is needed. At this point, we feel that reporting the finding is merited, and we hope that the reviewer agrees.

The following text has been added starting on line 437:

What we can conclude based on analytic and computational work is that randomly wandering leader cells in the presence of a cellular density gradient produce cluster migration, and that this migration is inhibited by participation of following cells. Ultimately, in all simulations shown, we found that deterministic forcing (as shown in Figure 3) never produces collective migration, under any conditions, while stochastic forcing (Figure 8) can.

***Reviewer remark:***

*1.20. Lines 450-451: Did the authors correct for their use of multiple comparisons, for example by performing a Bonferroni correction? If not, can they explain why?*

***Author Reply:***

We thank the reviewer for this suggestion. To our understanding, Bonferroni corrections illustrate a more accurate significance interval when multiple tests conducted suggest significant differences. Our analysis revealed no significant differences between Kelvin-Voigt and Standard Linear Solid. This result was desirable in that it indicates both are suitable models to describe the mechanism.

Our statistical analysis reveals no significant differences between groups, thus even with a Bonferroni correction, our results would still indicate no significant differences. The lack of significance showed that Kelvin-Voigt and Standard Linear Solid are both suitable models to describe this mechanism.

The following text has been revised on line 545:

Multiple Student t-test were performed on Kelvin-Voigt and Standard Linear Solid data. Our analysis revealed no significant differences between groups (p>0.05), indicating that both models are suitable for describing the proposed mechanism.

**REVIEWER #2**

***Reviewer remark:***

*2.1. The authors should explain how their mechanism differs from cohesotaxis (see references below) and cite the relevant literature on that.

Example cohesotaxis references:

Weber, G.F., Bjerke, M.A. and DeSimone, D.W., 2012. A mechanoresponsive cadherin-keratin complex directs polarized protrusive behavior and collective cell migration. Developmental cell, 22(1), pp.104-115.

Zhao, J., Cao, Y., DiPietro, L.A. and Liang, J., 2017. Dynamic cellular finite-element method for modelling large-scale cell migration and proliferation under the control of mechanical and biochemical cues: a study of re-epithelialization. Journal of The Royal Society Interface, 14(129), p.20160959.*

***Author Reply:***

We thank the reviewer for this helpful recommendation and insight into a relevant mechanism. While chemotaxis is quite closely related to what we are describing, our model contains key differences from what is described in cohesotaxis studies. We explore an asymmetrical cohesion strength within a cluster experiencing nonpolarized protrusions. No cell-substrate differences between front and back (i.e. net external traction stress) are involved, whereas previous studies explore a symmetrical cohesion within a cluster, monopolar protrusions, and integrin interactions with the substrate are key. The text has been updated to discuss our proposed mechanism as it relates to cohesotaxis.

The following text has been added starting on line 462:

We have demonstrated that a gradient in *internal* connection strengths within a cluster of cells is sufficient to produce directional migration. This mechanism shares features with cohesotaxis, in which cell clusters migrate due to increased *external* traction stress produced by leading cells (75-77). Fundamentally, cohesotaxis relies on an external stress gradient in the presence of symmetric cohesion between cells experiencing monopolar protrusions, while our analysis indicates that asymmetrical cohesion of cells experiencing nonpolarized protrusions in the presence of symmetric adhesion to the substrate can drive collective migration. Analogously, cohesotaxis is akin to a locomotive pulling a train, while this new mechanism is closer to metaboly, in which the Euglena propels itself following internal deformation.

***Reviewer remark:***

*2.2. The authors suggest a gradient in the spring stiffness throughout the tissue, but is that actually necessary? Would it be sufficient just to have the leading cell attached by a stiffer connection?*

***Author Reply:***

While it is possible that just the leading cell with a stiffer connection is sufficient, biologically speaking the local growth factor concentration is known to promote the upregulation of cadherin expression within individual cells, and the cells with higher levels of cadherin expression have the ability to form stronger cell-cell attachments. We model the gradient of growth factor concentration in the environment using a gradient of spring stiffness between cells. We have revised the text to clarify this point.

The following text has been revised starting on line 152:

In our model, both the strengths of repulsion and viscosity between adjacent agents are constant for all cells in a cluster. Tensile components of $K_{1}$ and $K_{2}$, however, are assumed to increase linearly along the length of the cluster. Biologically speaking, local growth factor concentration is known to promote the upregulation of cadherin expression within individual cells, and the cells with higher levels of cadherin expression enable stronger cell-cell attachments (48, 49). We model the gradient of growth factor concentration in the environmental using a gradient of spring stiffness between cells. This model is based on experimental data mentioned earlier (cf. Fig. 1) demonstrating that clusters of retinal progenitor cells exhibit cadherin-mediated chemotaxis in the presence of an FGF gradient (17, 19). This increase in tensile strength is intended to model a response to a gradient in an external factor. Individual cells in the higher growth factor concentration environment experience more upregulation of cell-cell adhesion proteins which lead to more stable and stronger adherens junction stability (48, 49). This differential cell-cell attraction strength will be referred to as the internal cohesion gradient herein.

***Reviewer remark:***

*2.3. Line 34 ingrate -> integrate*

***Author Reply:***

We thank the reviewer for pointing out this critical typo.

The following text has been revised on Line 44:

In this therapy, stem or progenitor cells, which can differentiate into the desired neuronal cell type, are inserted into a host tissue and must then migrate toward areas of damage, effectively integrate within the native environment, and form novel synaptic connections to recreate the neural pathway, restoring the damaged tissue.

***Reviewer remark:***

*2.4. Lines 104 & 105- rewrite; it appears the final term is described twice and the descriptions differ.*

***Author Reply:***

We thank the reviewer for pointing out this critical error. The discrepancy in the explanation of terms has been removed.

The following text has been revised on line 128:

Correspondingly we write $\eta\dot{\varepsilon}=\eta_{i}\dot{\varepsilon}_{i}+\eta_{e}\dot{\varepsilon}_{e}$, where $\eta_{i}\dot{\varepsilon}_{i}$ is a viscous stress that depends on relative velocities, $\dot{\varepsilon}_{i}$, of adjacent cells, and $\eta_{e}\dot{\varepsilon}_{e}$ depends on velocity of a cell in the lab frame.

***Reviewer remark:***

*2.5. Line 128- replace semicolon with comma*

***Author Reply:***

Thank you again for your careful reading. The semicolon was replaced with a comma as seen below.

The following text has been revised on line 153:

Tensile components of $K_{1}$ and $K_{2}$, however, are assumed to increase linearly along the length of the cluster.

***Reviewer remark:***

*2.6. Paragraph (lines 148-156)- There should be a better description of exactly what is done here. For example, what are the minimum and maximum cohesion strengths? What does cohesion strength mean (K_a?)? This is mentioned later, so if you do not want to define it here, then refer to the relevant section later in the paper. What does ‘relax’ mean? Do you integrate the equation on line 412/ line 414, and for how long? Precisely what parameter values are used.*

***Author Reply:***

We define the cohesion strength (Ka) as the attractive force between two neighboring cells in the methods section. We define relaxation as the time where the spring-damper system describing the intercellular interactions returns to a no stress state, which typically occurs when the cells are just touching. We solve the equations using the RK4 iterative method for every timestep between any stochastic movement. Exact parameter values are shared in the respective figure captions. We have updated the text to add clarity on these points.

The following text has been revised starting on line 181:

We begin with the case shown in Fig. 3(a), where N = 5 representative of a small cluster as in experiments of Fig. 1, simulated cells are initialized in a linear configuration from an initially relaxed state with no initial velocity and linearly increasing cohesion strength from bottom (*N_1_*) to top (*N_5_*), where the top cells’ cohesion are 10 times stronger than the bottom cells (additional key parameters are given in the caption Fig 3). Periodically, the two outermost cells (*N_1_* and *N_5_*) are shifted an equal distance away from the center of mass and given time to relax, where cells return to a zero-stress state (which typically occurs when they are just touching their neighbors), under the influence of cohesive interaction with their nearest neighbor (see methods).

And the following text has been revised on line 510:

The ODEs are solved using 4^th^ order Runge Kutta integration, for the duration of the “relaxation” time (the time period between stochastic events), where the spring damper systems move toward a no stress state.

***Reviewer remark:***

*2.7. Line 153- remove ‘least’*

***Author Reply:***

The authors thank the reviewer for this comment. We have rewritten the sentence to add clarity.

As mentioned above, the following text has been revised on line 188:

As expected, the cells with the strongest tensile moduli (near the leading edge of the cluster) deviate the least from the center of mass while the cells with the weakest tensile moduli (near the trailing edge) deviate the most from the center of mass. The center of mass of the cluster (dashed line), however, remains stationary.

***Reviewer remark:***

*2.8. Legend figure 3: define protrusion amplitude, period and gradient amplitude.*

***Author Reply:***

The definitions have been added to the caption.

The following text has been revised in the caption of Figure 3:

*Key parameters are as follows: gradient strength (ratio of strongest to weakest cohesion strengths) = 10, cluster size = 5, protrusion amplitude (length of protrusions) = 1, protrusion period (frequency of protrusions) = 15. Protrusion amplitude is a fixed value in this simulation, whereas all other simulations have a variable amplitude using a Gaussian whose standard deviation is the specified protrusion amplitude.*

***Reviewer remark:***

*2.9. Line 197 leading cell -> leading cells*

***Author Reply:***

The authors thank the reviewer for noticing this typo.

The following text has been revised on line 239:

For now, we analyze cluster motion in the limit that leading cells relax very rapidly and trailing cells relax very slowly.

***Reviewer remark:***

*2.10. Line 198- you say the lowest cell, but it looks like the highest cell. Should Fig. 5 a) and b) be flipped on a horizontal axis to agree with the descriptions… (Also line 202/203 upwards and downwards seem to differ from figure.)*

***Author Reply:***

The authors thank the reviewer for noticing this discrepancy. The text has been modified to match the figure.

The following text has been revised starting on line 240:

As depicted in Fig. 5(a), the leading cell in a cluster takes steps forward or back at stochastic distances defined by a Gaussian with standard deviation $\sigma$ indicated by dashes in the plots to the right of the cells. Our analysis is purely computational, but physiologically speaking this could represent the effect of pseudopod or adhesion formation (54-56).

And the following text has been revised starting on line 244:

If protrusions are short compared to the distance to the nearest cell, inward (extending the cell inward to the center of the cluster) and outward (extending the cell away from the cluster center) protrusions will be equally likely.

***Reviewer remark:***

*2.11. Line 272 Fig. 5(b) -> Fig. 6(b)*

***Author Reply:***

The authors thank the reviewer for noticing this mistake. The text has been corrected.

The following text has been revised on line 331:

This contrast can be evaluated, as shown in Fig. 6(b), where we plot longer simulations with and without stochastic extension, and with and without a cohesion gradient.

***Reviewer remark:***

*2.12. Paragraph (lines 288-296). If you reduce the velocities to zero after every timestep, then do the results not depend on your timestep? If not, please explain why? If so, this should be acknowledged- it seems unphysical.*

***Author Reply:***

To clarify, only in Kelvin-Voigt simulations did we reduce the velocities to zero to mimic a low Reynolds number in a viscous environment. The Standard Linear Solid model includes a term to describe viscous creep, so less damping is needed to mimic the same environment.

The following text has been revised starting on line 358:

All simulations are computationally damped to mimic a low Reynolds number in a viscous cellular environment. To achieve this computational damping, we consider the Stokes limit for KV simulations (i.e. we reduce all velocities by 100% after every timestep). SLS simulations include internal viscous creep which creates an additional damping, so we reduce velocities less (by 25%) every timestep for those simulations.

***Reviewer remark:***

*2.13. Line 372- The authors should put the work in context by discussing of other means of collective migration, especially those, such as cohesotaxis, which may be quite related.*

***Author Reply:***

The authors thank the reviewer for this comment. We have revised our conclusion section to include a discussion on previously established collective migration mechanisms including chemotaxis and cohesotaxis and how our model relates to them.

The following text has been revised in the Conclusion section:

Collective migration is a widely observed phenomenon across many cellular processes including morphogenesis, wound healing, cancer invasion, and tissue regeneration. Many different collective migration mechanisms have been proposed over the years including cell-cell and cell-substrate adhesion (50, 66), leader-follower dynamics (67, 68), contact inhibition of locomotion (69, 70), and gradient sensing (71-73). Many of these mechanisms work in tandem to generate effective collective motion and how they interact with one another is incompletely understood. Here we focus on the effects of cell-cell cohesion interactions produced by an external chemical gradient. We have performed a detailed kinematic analysis of cells in a cluster, and we have reached the following surprising findings. These differ from results reported for other collective phenomena.

1) Internal interactions within a cluster can contribute to net, external, motion of the cluster.
2) This effect requires individual cells to migrate stochastically: periodic displacements of cells do not produce net cluster motion.
3) The effect can arise when the leading cells are more closely spaced than the characteristic distance of stochastic migration. This spacing can be produced by an internal cohesion gradient.
4) The effect requires that migration of cells within a cluster be suppressed compared to migration of leading cells.

Our model has demonstrated a mechanism that explains why neural progenitors migrate along a growth factor gradient more effectively in clusters than as single cells. This observation challenges traditional understanding of chemotactic mechanisms in which individual cells sense their environment and growth factor binding triggers a signaling process guiding migration (74). We have demonstrated that a gradient in *internal* connection strengths within a cluster of cells is sufficient to produce directional migration. This mechanism shares features with cohesotaxis, in which cell clusters migrate due to increased *external* traction stress produced by leading cells (75-77). Fundamentally, cohesotaxis relies on an external stress gradient in the presence of symmetric cohesion between cells, while our analysis indicates that *internal* asymmetrical cohesion in the presence of environmental noise can drive collective migration. Analogously, cohesotaxis is akin to a locomotive pulling a train, while this new mechanism is closer to metaboly, in which the Euglena propels itself following internal deformation.

***Reviewer remark:***

*2.14. Line 385- the authors should add a reference to the end of the first sentence on this page.*

***Author Reply:***

The authors thank the reviewer for pointing out this oversight. Appropriate citations have been added to line 475.

***Reviewer remark:***

*2.15. Line 412- Surely this depends on whether the spring to n-1 or n+1 is under tension (i.e. Ka and Kr may be switched).*

***Author Reply:***

You are correct and we have added some text to clarify this.

The following text has been revised on line 504:

In both cases, the force on a particular agent is determined by the relative positions of the neighbors and whether the initial agent experiences tension (K_a_) or compression (K_r_), therefore the K terms can be switched (as seen in the following equations).

$\ddot{x}_{n} =\left[ \left( K_{r}x_{n-1}-\left( K_{r}+K_{a} \right)x_{n}+K_{a}x_{n+1} \right)- \eta\dot{x}_{n} \right]$	[7.2]

${\overset{⃛}{x}}_{n}=\frac{1}{\tau}\left( K_{r}x_{n-1}-\left( K_{r}+K_{a} \right)x_{n}+K_{a}x_{n+1} \right)+ \eta\dot{x}_{n}-\frac{1}{\tau}\ddot{x}_{n}$	[8.2]

***Reviewer remark:***

*2.16. Line 444 multiplied to -> multiplied by*

***Author Reply:***

We thank the reviewer for their careful reading. The text has been corrected.

The following text has been revised on line 536:

The environmental viscosity is an additional user defined parameter in our model and for computational simplicity, it is a value between 0 and 1 that is multiplied by the initial velocity (and acceleration) prior to the numerical calculations.

**REVIEWER #3**

***Reviewer remark:***

*3.1. Abstract: The authors introduce the important concept of “asymmetry near the leading cell” but it is not clear at this point what asymmetry they are referring to. The authors should clarify, “..asymmetry [of what?]…” in this key sentence.*

***Author Reply:***

The authors thank the reviewer for this question. Here we are referring to the asymmetry of the cohesive attachment strengths near the leading edge of a cluster compared to the trailing edge of a cluster.

The following text has been revised on line 12:

Unlike other swarming phenomena, we find that this effect is driven by cohesion asymmetry near the leading cell, and motion of interior cells contribute minimally – and in fact interfere with – a cluster’s persistent migration.

***Reviewer remark:***

*3.2. Introduction: A related point to the one above, is that the authors should relate the concept of “internal cohesion gradient” to the biology of cell-cell adhesion. Could the internal cohesion gradient, which is a lynchpin of the model, represent gradients of cadherin receptors in neighboring cells? Or gradients of activated cadherin receptors across neighboring cells? If this is the case, the authors should try to cite literature that reports such biological observations because that will help demonstrate the biological relevance of their model.*

***Author Reply:***

We refer to the internal cohesion gradient as differential cadherin receptor expression that develops in individual cells in response to their local environmental growth factor concentration, and the higher expression of cadherin molecules in the individual cells leads to a stronger attachment to their neighbors. This upregulation of cadherins in response to growth factors was observed by our group and others. We’ve revised the text to address this question.

The following text has been revised starting on line 152:

In our model, both the strengths of repulsion and viscosity between adjacent agents are constant for all cells in a cluster. Tensile components of $K_{1}$ and $K_{2}$, however, are assumed to increase linearly along the length of the cluster. Biologically speaking, local growth factor concentration is known to promote the upregulation of cadherin expression within individual cells, and the cells with higher levels of cadherin expression enable stronger cell-cell attachments (48, 49). We model the gradient of growth factor concentration in the environmental using a gradient of spring stiffness between cells. This model is based on experimental data mentioned earlier (cf. Fig. 1) demonstrating that clusters of retinal progenitor cells exhibit cadherin-mediated chemotaxis in the presence of an FGF gradient (17, 19). This increase in tensile strength is intended to model a response to a gradient in an external factor. Individual cells in the higher growth factor concentration environment experience more upregulation of cell-cell adhesion proteins which lead to more stable and stronger adherens junction stability (48, 49). This differential cell-cell attraction strength will be referred to as the internal cohesion gradient herein.

***Reviewer remark:***

*3.3. Introduction: (Page 2, last paragraph): The sentence that begins, “In our lab we study…” seems out of place in a manuscript and reads more like a grant. My suggestion is to begin this sentence with: “We have previously shown in vitro that in the presence…”*

***Author Reply:***

Thank you for this useful suggestion.

The following text has been revised on line 51:

We have previously shown *in vitro* that in the presence of a fibroblast growth factor (FGF) signaling gradient, individual retinal progenitor cells (RPCs) migrate seemingly randomly, while clusters of cells under the same conditions exhibit persistent motion toward the gradient maximum (17, 18).

***Reviewer remark:***

*3.4. Introduction: The reproduction of Figure 1 from a different published paper may not be allowed by the journal and I think it would be better to just cite the paper where this finding was published.*

***Author Reply:***

We want to clarify that this figure was developed by the authors and is completely original. It was generated to show 1D trajectories from the 2D data that was published in the associated reference.

***Reviewer remark:***

*3.5. Introduction: (Page 3, first paragraph): The sentence that begins: “These factors do not appear to affect persistent cell migration of individual cells,…” is confusing. What do “these factors” refer to in this sentence? Cadherins or growth factors?*

***Author Reply:***

We are referring to growth factors here. We have updated the text to provide clarification.

The following text has been revised starting on line 66:

Intriguingly, the growth factors examined in both fly and mouse RPCs upregulate the expression of cadherins (19-21): trans-membrane proteins that mediate cell-cell cohesion. These growth factors do not appear to affect persistent migration of individual cells; hence we investigate here the mechanism by which internal, cell-cell, interactions affect net motion of a cluster.

***Reviewer remark:***

*3.6. Introduction: (Page 3, last paragraph): The authors state that “…cells can generate an external force that individual cells do seem to not feel.” This is confusing. How do we know that cells don’t “feel” an external force? Maybe it would help to define what is meant by “external force”.*

***Author Reply:***

The external forces we are referring to are those that arise from cell-cell connections which only occur in clusters and will not be seen in individually migrating cells. We have edited the text to provide this clarification.

The following text has been revised on line 79:

Some headway into resolving the paradox that interactions between cells connected within a cluster can generate an additional external force (arising from cell-cell connections) that individual cells do not feel can be made by observing that a gradient in internal cell-cell cohesion breaks the symmetry between what we’ll term the “front” (higher FGF) and “back” (lower FGF) of a collection of cells.

***Reviewer remark:***

*3.7. Introduction: (Page 4, last paragraph): This is the first place where the authors introduce some key assumptions of their model, and I think they should expand on the discussion of the model assumptions and limitations throughout the Results, as well. One major assumption stated here is that substrate stresses are assumed to be identical along the length of the cluster. The authors should justify this assumption and/or explain whether this is a limitation of their model, given what has been experimentally observed in biology. Also, what does “omnidirectional wandering” mean?*

***Author Reply:***

In our work, we focused on the cell-cell interactions and found that the mechanism can be independent from cell-substrate interaction. In our application, retinal stem cell transplantation, this is particularly useful as the cells are migrating within an environment constantly experiencing degeneration which disrupts cell-matrix interactions. In addition, omnidirectional wandering is another way of stating pure stochasticity with unbiased directional movement. We have updated the text to provide this clarity.

The following text has been revised on line 101:

We emphasize that substrate stresses are assumed to be identical along the length of the cluster: the net force toward increased gradient emerges only from asymmetry of internal cohesive forces combined with stochastic wandering. This is potentially important for future applications of retinal transplantation, as the environment associated with degenerative disorders of the eye can result in dysregulation of cell-matrix connections (38).

***Reviewer remark:***

*3.8. Results: (Page 7, first paragraph): This sentence is confusing: “…however if either mechanism is activated periodically in time using alternating for-ward and rear-ward distances periodically in time, no collective motions of clusters is seen…” What does “periodically in time” refer to? What does “alternating for-ward and rear-ward distances” refer to? Are these distances set lengths? What is the period of time during which the alternating is occurring? Also, what does “migrate stochastically” mean? Does an individual cell randomly decide to move forward and backward according to some period of time that is set in the model, or is that period of time stochastic, as well?*

***Author Reply:***

Periodically in time refers to a consistent amount of time where the outermost cells experience an intrinsically generated movement. Alternating forward and reward distances refer to the direction that the outermost cells are moving. We have updated the terms to outward and inward to provide more clarity on their directions. In the case of figure 3, these distances are one set length, in all other figures they are determined by a mean 0 gaussian distribution. The period of time is not stochastic in all cases.

The following text has been revised on line 171:

However, if migration is deterministically activated (i.e. cells moving in alternating inward and outward directions periodically in time), no collective motion of clusters is seen in our simulations. On the other hand, if the same migration of individual cells is invoked stochastically (i.e. cells moving in either direction randomly), persistent forward motion of clusters emerges.

***Reviewer remark:***

*3.9. Results: Most of the results sub-heading titles are confusing and do not adequately describe the results that are being presented in that sub-section. For example, what does “Baseline” refer to in the 2nd sub-heading title? Similarly, what does “Analysis: baseline case plus diffusion of out cells” mean (specifically, what is being analyzed?). Also, “Quantification” as a sub-heading is confusing. (Quantification of what? And were the prior results not also “quantification”?). And, “Qualitative confirmation” (of what?). And, “Quantitative parameteric test” (of what?). Revising the Results sub-section headings will help improve clarity and help the reader better understand how each section builds (or relates to) the prior results.*

***Author Reply:***

The authors thank the reviewer for this critical comment. The subheading in the results section have been updated to include more specific details and add clarity.

The following subheadings have been revised:

**Deterministic Model: Differential cohesion alone does not produce cluster motion**

**Analysis: deterministic case vs. stochasticity on the outermost cells**

**Quantification: mathematical derivation of cluster mean displacement**

**Qualitative confirmation: cluster migration with and without an internal gradient**

**Quantitative testing of key parameters: gradient strength, cluster size, protrusion amplitude, and protrusion period**

***Reviewer remark:***

*3.10. Results: (Page 7, last paragraph): The sentence: “As expected, the cells with the strongest (weakest) tensile moduli deviate least (most) from the least center of mass…” is confusing.*

***Author Reply:***

The authors thank the reviewer for this comment. We have rewritten the sentence to add clarity.

The following text has been revised on lines 188-191:

As expected, the cells with the strongest tensile moduli (near the leading edge of the cluster) deviate the least from the center of mass while the cells with the weakest tensile moduli (near the trailing edge) deviate the most from the center of mass. The center of mass of the cluster (dashed line), however, remains stationary.

***Reviewer remark:***

*3.11. Results (Figure 7 and Page 15, last paragraph): The authors inconsistently refer to “Gradient strength ratio” and “Gradient strength” in the figure caption and in the text. Do these terms mean the same thing? Also, it should be clear to the reader that the authors are referring to the “cohesive gradient strength” or “cohesive gradient strength ratio”, and these terms should be clearly defined in the manuscript.*

***Author Reply:***

The authors thank the reviewer for noticing this discrepancy. The gradient strength is defined as the ratio of the strongest and weakest cohesion strength; therefore, they do mean the same thing. We have removed the references to gradient strength ratio in the caption in order to maintain consistency and add clarity.

***Reviewer remark:***

*3.12. Results: Why did the authors initially chose to represent 5 cells in their model?*

***Author Reply:***

The authors thank the reviewer for this question. The answer is we choose this number to represent a small cluster size similar to the experimental results shown in Figure 1, where small clusters were defined as 5 to 15 cells. We also chose a smaller N to reduce the computational run times.

The following text has been revised on line 181:

We begin with the case shown in Fig. 3(a), where N = 5, representative of a small cluster as in experiments of Fig. 1, simulated cells are initialized in a linear configuration from an initially relaxed state with no initial velocity and linearly increasing cohesion strength from bottom (*N_1_*) to top (*N_5_*).

***Reviewer remark:***

*3.13. Results: (Page 18, first paragraph): The authors state that the model makes the surprising prediction that the mechanism of cluster migration that they have explored depends on “suppressing internal cellular motion”. This is based on the fact that when they reduce the Gaussian standard deviation of the central cells by 75% net cluster migration is rescued. What happens when they reduce it by even more (like 95%) or by less (like 1%)? This is an important prediction and showing a full sensitivity analysis would help the reader understand how significant this effect is on cluster migration speed.*

***Author Reply:***

The authors thank the reviewer for this comment. In figure 8 we show the comparison between the center cells being suppressed 100% (green), 0% (red), and 75% (blue). Reducing them by 95% would be quite similar to the green data set and reducing them by 1% would be similar to the red data set.

***Reviewer remark:***

*3.14. Conclusion: Revisiting my comment #2 above, I think the impact of the paper would be elevated by discussing whether cadherin expression (and/or activation) gradients across neighboring cells have been examined and how that data relates to the “cohesive gradient strength” which is the central prediction of this model.*

***Author Reply:***

The authors thank the reviewer for this comment. We have revised the text in the conclusion to include more discussion on the impact of the cadherin expression on the impact of retinal transplantation.

The following text has been revised starting on line 471:

This novel understanding of collective migration is essential to progressing research in cell replacement therapy. It has been well established that cell-cell adhesion molecules can play a critical role in morphogenesis and migration in tissue regeneration. Previous studies have shown an upregulation of cadherins by external growth factors as well as an increase in the directionality of cellular migration (17, 19). Our findings indicate that an internal cohesion gradient, stemming from differential external factors, leads to collective migration not previously described. We propose that improved understanding of this new mechanism may support future replacement cell transplantation therapies.
