# Supplementary material for: Internal cohesion gradient as a novel mechanism of collective cell migration
Source: PLoS Comput Biol. 2025 Mar 10;21(3):e1012769. doi: 10.1371/journal.pcbi.1012769 (PMC12077783; doi:10.1371/journal.pcbi.1012769)
Supplement: Attachment — Submitted filename: PLOS - Reply to Reviewers 2.docx [file pcbi.1012769.s007.docx]

**Maribel Vazquez, Sc.D.**

**Professor of Biomedical Engineering**

**599 Taylor Road, BME-219**

**Piscataway, NJ 08854-5610**

<https://www.vazlab.org/>

January 2, 2025

Dr. Feilim Mac Gabhann and Dr. Jason Papin

Editors-in-chief, PLOS Computational Biology

1265 Battery Street, Suite 200

San Francisco, CA 94111

United States

Dear Dr. Gabhann and Dr. Papin,

I write to attach author revisions to the manuscript, ‘***Internal Cohesion Gradient as a Novel Mechanism of Collective Cell Migration.***’ PCOMPBIOL-D-24-00775. The reviewers’ remarks have helped us greatly enhance this manuscript. Each reviewer comment is addressed point-by-point below and highlighted in red text within the revised manuscript.

Please advise if we can provide any further information to aid in the review process.

Thank you.

Sincerely,

____________________________

Dr. Maribel Vazquez

Professor and Principal Investigator

Department of Biomedical Engineering

Rutgers University, The State University of New Jersey

599 Taylor Road, BME-219

Piscataway, NJ 08854-5610

Office: 848.445.6568 Fax: 732.445.3753

<https://www.vazlab.org/>

---------------------------------------------------------------------------------------------------------------------

**REVIEWER #1**

***Reviewer remark:***

*1. Relevant MATLAB codes have been provided, but the raw data that have produced the figures has not been sent to the reviewers and there is no information on how to obtain them. The authors should upload the raw data to a repository (e.g., Dryad) and cite a link to the dataset in the manuscript.*

***Author Reply:***

We thank the reviewer for this reminder. The raw data seen in Figure 1 is publicly available in the supplemental information of the associated PLOS paper (reference #17). Here is the DOI for your reference: <https://doi.org/10.1371/journal.pone.0226250>

The following text has been revised in the caption of Figure 1:

*Each plot shows 3 trials (raw data provided in Pena 2019 supplemental information (17)); distances traveled are normalized to the number of cells in the cluster.*

***Reviewer remark:***

*2. Lines 192-193 and Lines 220-221: What happens to the center of mass when the outermost cells are (deterministically) perturbed at different frequencies? It seems that if the uppermost cell were perturbed at a higher frequency than the lowermost cell, then this could result in a net migration. Have the authors tested this case as well?*

***Author Reply:***

We thank the reviewer for this question. If we perturbed the outmost cells at different frequencies, we do find that the cluster will migrate toward the direction of higher frequency perturbations (see figures below). However, biologically speaking, we are looking at a homogenous cluster of a single cell type and therefore we made the assumption that the frequency of protrusion formation would be equal across all individual cells.

***Reviewer remark:***

*3. Figure 6(b): It seems that the persistent movement is only beginning to emerge at the final time in these graphs. The authors should run this computation for a longer time range to confirm that this is truly a persistent trajectory and not, say, a fluctuation like in the case of the blue line, which has amplitude of the same order of magnitude but returns to the initial position. Including information about the range of values across all 100 runs would also be informative.*

***Author Reply:***

We thank the reviewer for this comment. Figure 6b was intended to serve as an example of possible trajectories clusters with and without gradients can make, highlighting that while clusters without a gradient may move considerably the net movement rests around zero. This is further confirmed by the linear trajectory in the MSD plot 6c. We have recreated the figure with a longer timescale to add clarity. In addition, we have added information about the range of values for panel c in the figures caption.

The following text has been revised in the caption of Figure 6:

*(c) Mean Squared Displacement analysis of N = 100 simulations of cell clusters without a gradient (blue) and with a gradient (red). Final positions range from -1 to 7 radii away from the initial position for clusters with a gradient vs ±3 radii away for clusters without a gradient. Cyan dashed lines indicate linear regression:* $y=\left( 231\pm0.2 \right)\times{10}^{-6}*x$*and quadratic regression:* $y=\left( 275\pm0.5 \right)\times{10}^{-6}*x-\left( 86.8\pm0.9 \right)*{10}^{-10}*x^{2}$*. Key parameters are as follows: gradient strength = 10, cluster size = 5, protrusion amplitude = 1, protrusion period = 15.*

***Reviewer remark:***

*4. Figure 6(c): It would be helpful to include the standard deviation of the MSD across all runs, in addition to the mean values. Also, can a linear and quadratic reference line be added to this plot?*

***Author Reply:***

We appreciate this suggestion and have incorporated linear and quadratic best fit lines to figure 6c and included information about the standard error in the caption.

The following text has been revised in the caption of Figure 6:

*(c) Mean Squared Displacement analysis of N = 100 simulations of cell clusters without a gradient (blue) and with a gradient (red). Final positions range from -1 to 7 radii away from the initial position for clusters with a gradient vs ±3 radii away for clusters without a gradient. Cyan dashed lines indicate linear regression:* $y=\left( 231\pm0.2 \right)\times{10}^{-6}*x$*and quadratic regression:* $y=\left( 275\pm0.5 \right)\times{10}^{-6}*x-\left( 86.8\pm0.9 \right)*{10}^{-10}*x^{2}$*. Key parameters are as follows: gradient strength = 10, cluster size = 5, protrusion amplitude = 1, protrusion period = 15.*

***Reviewer remark:***

*5. Line 236: There appears to be a typo here (protrusion rather than protrusions).*

***Author Reply:***

We thank the reviewer for their careful reading; corrected in the revised manuscript.

The following text has been revised on line 236:

This allows direct comparison with Fig. 3 and agrees with recent findings that leader cell specification emerges from the existence of an unconstrained free edge which enables extended protrusion formation.

***Reviewer remark:***

*6. Line 469: No background has been given on Euglena organisms to this point. This reference needs to be put into more context.*

***Author Reply:***

We thank the author for this comment. We have revised the text below for clarity.

The following text has been revised on line 472:

Analogously, cohesotaxis is akin to a locomotive pulling a train, while this new mechanism

is closer to metaboly, in which organisms propel itself following internal

deformation.
